# *Fasciola hepatica* Soluble Antigen (*Fh*Ag)-Induced NETs Under Hypoxic Conditions Exert Cytotoxic Effects on Hepatic Cells In Vitro

**DOI:** 10.3390/ani14233456

**Published:** 2024-11-29

**Authors:** Tamara Muñoz-Caro, Pamela Quiroz, Cristina Abarca, Marcela Gómez-Ceruti, Pablo Alarcón, Stefanie Teuber, Max Navarro, Anja Taubert, Carlos Hermosilla, Rafael A. Burgos

**Affiliations:** 1 Escuela de Medicina Veterinaria, Facultad de Medicina Veterinaria y Recursos Naturales, Universidad Santo Tomás, Talca 3460000, Chile; 2 Centro de Investigación de Ovinos Para El Secano OVISNOVA, Facultad de Medicina Veterinaria y Recursos Naturales, Universidad Santo Tomás, Talca 3460000, Chile; 3 Laboratory of Inflammation Pharmacology, Institute of Pharmacology and Morphophysiology, Faculty of Veterinary Sciences, Universidad Austral de Chile, Valdivia 5090000, Chilerburgos1@uach.cl (R.A.B.); 4 Institute of Veterinary Clinical Science, Faculty of Veterinary Sciences, Universidad Austral de Chile, Valdivia 5090000, Chile; 5 Institute of Parasitology, Faculty of Veterinary Medicine, Justus Liebig University Giessen, 35392 Giessen, Germanycarlos.r.hermosilla@vetmed.uni-giessen.de (C.H.)

**Keywords:** *Fasciola hepatica*, neutrophil extracellular traps, NETosis, ROS, hypoxia, cytotoxicity, MMP-9, innate immunity, sheep, livestock

## Abstract

*Fasciola hepatica* is a parasitic trematode that causes fasciolosis in sheep, provoking a decrease in their reproductive capacity, weight gain, meat and milk production, and wool quality. In the pathogenesis of *F. hepatica*, the penetration and migration of parasitic stages through the liver provoke intense inflammatory immune responses and tissue damage. The aim of this study was to investigate the cytotoxic effects of *Fascila hepatica*-induced ovine NETs in exposed hepatocytes in vitro, and to analyze whether *F. hepatica* antigens (*Fh*Ag) trigger the release of ovine NETs under hypoxic conditions as well as the roles of matrix metalloproteinase-9 (MMP-9) and CD11b in this cellular process in vitro. Here, isolated ovine PMNs were co-cultured with *Fh*Ag under hypoxia (5% O_2_) and NETs were visualized via immunofluorescence analyses, confirming their classical characteristics. The quantification of NETs in response to *Fh*Ag in hypoxic conditions significantly enhanced the formation of anchored and cell-free NETs (*p* < 0.01), and NADPH oxidase (NOX) inhibitor diphenylene iodonium (DPI) significantly reduced their production (*p* < 0.05). Furthermore, the cytotoxic effect of NETs on hepatic cells was determined by using a live/dead-staining with Sytox Orange, thereby demonstrating that *Fh*Ag-induced NETs are cytotoxic for hepatic cells (*p* = 0.001). We additionally analyzed PMN supernatants to determine the enzymatic activity of MMP-9, observing that *Fh*Ag exposure enhances MMP-9 release in ovine PMNs (*p* < 0.05) but not in bovine PMNs. Interestingly, by using flow cytometric analysis, we determined that the exposure of PMNs to *Fh*Ag does not increase the CD11b surface expression of ovine PMNs. This could be an effect of the activation of other surface receptors or transcription factors involved in *F. hepatica*-induced NETosis. Consequently, we hypothesize that *F. hepatica*-induced NETs play a role in the pathogenesis of fasciolosis, contributing to liver tissue damage if released in an uncontrolled manner.

## Simple Summary

Fasciolosis is one of the parasitic diseases that exerts the greatest impact on sheep productive systems worldwide. Affected sheep generally show a decrease in their reproductive capacity, weight gain, meat and milk production, and wool quality. In the pathogenesis of *Fasciola hepatica*, the penetration and migration of parasitic stages through the liver provoke intense inflammatory immune responses and tissue damage. Following strong activation signals, polymorphonuclear neutrophils (PMNs) reportedly release chromatin and granular proteins into the extracellular space, forming DNA traps called neutrophil extracellular traps (NETs). It was recently demonstrated that *F. hepatica* induces NET formation in vivo in the liver parenchyma from naturally infected sheep. Thus, in this paper we investigated *F. hepatica*-mediated NET-derived cytotoxic effects in exposed hepatic cells in vitro as well as *F. hepatica*-triggered ovine NETs under hypoxic conditions (5% O_2_). Our results confirm the ability of ovine PMNs to form NETs in low-oxygen environments after *F. hepatica* antigen (*Fh*Ag) stimulation, as seen in many pathological and pro-inflammatory states, and their cytotoxic effects on exposed hepatic cells. In addition, we demonstrate that this defence mechanism is NADPH oxidase (NOX)-dependent and therefore a tightly regulated molecular process. Overall, we hypothesize that NET formation plays a role in the pathogenesis of fasciolosis, and NETs contribute to liver tissue damage if released in an uncontrolled manner.

## 1. Introduction

*Fasciola hepatica* is a parasitic trematode that provokes fasciolosis, also called the liver fluke disease, affecting a wide range of vertebrate species, especially sheep, in temperate regions of the world [1]. Additionally, human fasciolosis is considered a neglected parasitic disease [2]. It is recognized by the World Health Organization (WHO) that 180 million people are at risk of infection [3]. In livestock animals, this parasite causes economic losses greater than US $3 billion per annum [4]. These economic losses are associated with the liver damage caused by juvenile trematodes migrating through the definitive hosts, resulting in poor food conversion, impaired fertility, and reduced wool and milk production [5]. It has been shown that the intensive and inadequate use of antiparasitic drugs that act against *F. hepatica* has resulted in the development of drug resistance [6]. Their use does not prevent the severe hepatic damage produced by later reinfections [7]; likewise, vaccines are not yet available [8]. Therefore, the development of novel control methods and therapeutic alternatives for fasciolosis is urgent needed [9]. Thus, a better understanding of host–parasite interactions is expected [10] to further refine the development of novel control strategies against *F. hepatica* infections [1].

In the pathogenesis of *F. hepatica*, juvenile parasites migrate through the liver parenchyma to reach the bile ducts, where they mature into adult flukes [11] and become blood feeders [12], completing their development and producing eggs that will be passed onto the pastures [1,12]. The migration of *F. hepatica* juvenile stages causes extensive tissue damage, resulting in hepatic pathogenesis (parasitic hepatitis) associated with acute fasciolosis [13]; it is particularly problematic in sheep when the massive migration of immature (juvenile) flukes into the liver occurs. Clinical signs of infection include anemia, ascites and abdominal pain, which are likewise related with sub-acute disease characterized by severe hepatic hemorrhage, leading in some cases to sudden death [14,15]. In sheep, a highly susceptible ruminant species, the parasite-induced migratory tracts in liver parenchyma are surrounded by immunocompetent cells, resulting in inflammation and the formation of granulomas [16]. In contrast, fasciolosis in cattle causes more extensive fibrosis and less visible tracts. This is thought to play a role in the partial resistance to reinfection but can progress to cirrhosis of the liver in severe cases [17]. In both cases, established parasites in the major bile ducts are long-lived, causing chronic cholangitis [4]. However, in the case of liver fluke infections in the field, animals can be continually reinfected, perpetuating the damage and eventually compromising overall liver function [16,18]. In addition, in severe infections, extensive damage can cause some parasite eggs to leak out into the liver parenchyma, leading to inflammatory granulomatous responses [19]. Interestingly, during fasciolosis, the flukes tolerate the low oxygen levels in the liver and the bile [20] and activate genes involved in anaerobic glycolysis, allowing the fluke to be a facultative anaerobe trematode [21].

The first line of control against *F. hepatica* invasion is provided by innate immune leukocytes such as polymorphonuclear neutrophils (PMNs), eosinophils, monocytes, and macrophages. These leukocytes become activated upon their first encounter with migrating stages of *F. hepatica* through pathogen recognition receptors (PRRs) that bind to parasite-specific molecules, aiming to hamper parasite development within the host [22,23]. Additionally, PMNs are the most abundant leukocytes circulating in the blood/lymph system and playing a crucial role in the development of innate immune and adaptive immune responses [24] via phagocytosis, the secretion of pro-inflammatory cytokines/chemokines, the production of reactive oxygen species (ROS), and the degranulation of antimicrobial peptides and proteins [25,26,27]. Additionally, the formation of neutrophil extracellular traps (NETs) is a more recently described defence mechanism of PMNs that is used to fight invading pathogens [28,29], including protozoan and metazoan parasites [26,30]. NETs are composed of fibrous materials containing decondensed chromatin, to which various cytoplasmic proteins are attached, such as neutrophil elastase (NE), myeloperoxidase (MPO), PAD4-citrunillated histones (H1, H2A-H2B, H3, and H4), gelatinase, pentraxin, and cathelicidin, among others [31,32,33]. So far, few reports exist in the literature on trematode-induced NETosis, i.e., *Schistosoma japonicum* [34], *Opisthorchis viverrine* [35], and *Fasciola gigantica* [36]. NET formation against *F. hepatica* has been demonstrated in cattle. In this species, it produces a rather weak reaction, with low levels of ROS generation [37]. Meanwhile, NET formation, induced by the same parasite in sheep, was recently demonstrated [38], showing that ovine PMNs release NETs in response to *F. hepatica* in vitro, with ROS generation and chemotaxis [38] therefore demonstrating differences in the innate immune responses of cattle and sheep [38]. More importantly, NETosis in response to *F. hepatica* in sheep has also been demonstrated in vivo in the liver parenchyma of naturally *F. hepatica*-infected sheep [38], indicating that NET formation might have a role in the pathogenesis of fasciolosis in vivo. In this context, it has been demonstrated that NETs may function as double-edged swords, serving as effective antimicrobial/antiparasitic defence mechanisms, but also as putative sources of molecules with immune effects and/or pro-inflammatory properties able to promote tissue and organ damage [39]. Therefore, this study aimed to analyze the tissue damage provoked by ovine NETs produced by *Fh*Ag in exposed hepatic cells, the effect of hypoxic conditions (5% O_2_) on NET release, and the role of gelatinase granules (MMP-9) and the CD11b receptor in the ovine innate immune response against this parasitic trematode.

## 2. Materials and Methods

### 2.1. Isolation of PMNs

For PMN isolation, healthy adult Suffolk sheep (*n* = 4) were bled by puncturing the jugular vein and peripheral blood was collected in BD Vacutainer^®^ citrate tubes. Then, approximately 20 mL of blood was diluted in 20 mL of sterile PBS with 0.02% EDTA (Sigma-Aldrich, St. Louis, MO, USA). This was layered on top of 12 mL of Ficoll^®^ separating solution (density = 1.077 g/L; Sigma-Aldrich) and the solution was centrifuged (800× *g*, 45 min). After the removal of plasma and peripheral blood mononuclear cells (PBMCs), the cell pellet was suspended in sterile Hanks’ Balanced Salt Solution (HBSS; 0.4 mM KH_2_PO_4_, 0.3 mM NaH_2_PO_4_, 136 mM NaCl, 6 mM glucose, 5 mM KCl, 0.9 mM CaCl_2_, pH 7.4) and ovine erythrocytes were lysed by using an hypotonic lysis solution composed of phosphate-buffered water (5.5 mM NaH_2_PO_4_, 8.4 mM HK_2_PO_4_, pH 7.2) for 1 min at room temperature (RT). This was repeated twice. After returning to isotonicity using a hypertonic phosphate buffer (5.5 mM NaH_2_PO_4_, 8.4 mM HK_2_PO_4_, 0.46 M NaCl, pH 7.2), cells were centrifuged at 600× *g* for 10 min and the pellet was washed twice with sterile HBSS at 600× *g* for 10 min. Finally, PMNs were resuspended in sterile HBSS, counted in a Neubauer haemocytometer chamber, and left on ice to rest (30 min) before use. For the isolation of bovine PMNs, three clinically healthy Holstein Friesian heifers (*n =* 3) were used and PMNs isolation was performed according to [40]. Briefly, heifers were bled by puncturing the jugular vein, and blood was collected in tubes with acid citrate dextrose (Becton Dickinson, Franklin Lakes, NJ, USA). The tubes were centrifuged at 1000× *g* at 20 °C for 20 min, the plasma and buffy coat were discarded, and the remaining cells were suspended in sterile HBSS. Erythrocytes lysis and the subsequent centrifugations were performed as indicated for ovine PMN isolation. In all experiments, animal handling was performed in accordance with the recommendations of the National Research and Development Agency (ANID) of Chile, which were performed according to the current Chilean Animal Protection Laws. This was endorsed by the ethical committee of Universidad Santo Tomás (permit number 130/2020) and Universidad Austral de Chile (permit number 519/2023).

### 2.2. Soluble Fasciola hepatica Antigen (FhAg) Preparation

For the preparation of soluble *Fh*Ag, the protocol of [37] was followed: four alive *F. hepatica* adults, collected from the liver of naturally infected cattle, were collected at a local butchery and immediately transported at 4 °C to the Laboratory of Veterinary Parasitology of Universidad Santo Tomás, Talca, Chile. They were immediately frozen in liquid nitrogen. Thereafter, frozen parasites were ground in a previously UV-sterilized cooled mortar (−80 °C for 1 h) for soluble *Fh*Ag preparation. Thus, 300 μL sterile phosphate-buffered saline (PBS; 1X; Sigma-Aldrich) was added into a mortar and the collected parasites were added and ground. The suspension was then sonicated in an ice bath with a digital ultrasonic bath Biobase UC-20A^®^ sonicator for five cycles of 15 s. The sonicated material was then centrifuged at 10,000× *g* for 20 min at 4 °C. The protein concentration of supernatant was measured using the method of BCA^®^ protein assay (Thermo Fisher Scientific, Waltham, MA, USA) according to the manufacturer’s instructions and final *Fh*Ag solutions were stored at −20 °C until further use.

### 2.3. Determination of Matrix Metalloproteinase 9 (MMP-9) Activities in PMN Supernatants

Given that the tertiary granule contents of mammalian PMNs, including MMP-9, contribute to tissue damage and to NET formation via their antimicrobial peptide/protease activities, as described elsewhere [27,31], and as MMP-9 activity against *Fh*Ag has not been assessed in either ovine or bovine species, here, we used PMNs from both ruminant species to elucidate possible differences among species. Thus, ovine PMNs (*n* = 4; 10^6^/500 μL HBSS/0.9 mM CaCl_2_) and bovine PMNs (*n* = 3; 10^6^/500 μL HBSS/0.9 mM CaCl_2_) were exposed to *Fh*Ag (100 μg/mL) for 15 and 30 min at 37 °C. For positive controls, the stimulation of PMNs with PAF (100 nM, 5 min, 37 °C) was performed, and PMNs seeded in sterile RPMI medium (Sigma-Aldrich) were used for negative controls. After incubation, cells were centrifuged (600× *g*, 6 min) and the supernatants were recovered for the assessment of gelatinase activity by zymography. Here, substrate gel electrophoresis was performed using the method described by [41]. Briefly, 10 μL of supernatant/slot was loaded on polyacrylamide gels (10%, 0.75 mm thickness) containing 0.22% of gelatin (Sigma-Aldrich). In parallel, a recombinant MMP-9 standard (Sigma-Aldrich M8945) and a molecular mass marker (Fermentas International Inc., Burlington, ON, Canada) were loaded as reference samples. The gels were run at 200 V for 1 h in a Bio-Rad Mini Protean II^®^ chamber (Bio-Rad Laboratories, Plano, TX, USA). Thereafter, the gels were incubated twice in Triton X-100 (2.5%, under constant shaking, RT, 30 min) and overnight at 37 °C in a reaction buffer (100 mM Tris, 200 mM NaCl, 10 mM CaCl_2_, pH 7.5). Then, gels were stained in Coomassie Brilliant Blue solution R-250^®^ (Winkler, 0.5% in acetic acid:methanol:water = 1:3:6) and MMP-9 (gelatinase B) enzymatic activity in the samples was determined according to the degree of gelatin degradation (visible as clear bands of 82 kDa) relative to the MMP-9-control by means of band intensity measurements, applying Image Studio Software v154e.

### 2.4. Immunofluorescence Microscopy Analyses for Visualization of FhAg-Induced NETosis Under Hypoxia (5% O_2_) In Vitro

The analysis of NET formation induced by *Fh*Ag was also performed via immunofluorescence microscopy analyses. Here, ovine PMNs (*n* = 4) added to a sterile serum-free RPMI 1640 cell culture medium (Sigma-Aldrich) without phenol red were seeded on sterile Millicell EZ^®^ slides (Millipore, Darmstadt, Germany), coated with poly-_L_-lysine (0.01%; Sigma-Aldrich) beforehand. Then, ovine PMNs (2 × 10^5^ PMNs) were stimulated with 100 µg/mL of *Fh*Ag according to [37]. We used a plain medium as a negative control and calcium ionophore A23187 (5 µM; Sigma-Aldrich) as a positive control at 37 °C hypoxic conditions (5% O_2_, 94% N_2_, 5% CO_2_) for 3 h in a hypoxia incubator chamber (Stemcell Technologies, Vancouver, BC, Canada). Afterwards, cells were fixed with 4% (*w*/*v*) paraformaldehyde (Merck, Burlington, MA, USA) for 20 min at RT, washed thrice with PBS, and stored at 4 °C until further use. Then, cells were analyzed for the detection of citrunillated pan-histones by using anti-histones (H1, H2A/H2B, H3, H4), antibody MAB3422 (Sigma-Aldrich; 1:200), and anti-NE antibody (AB68672 Abcam, Cambridge, UK; 1:200). They were incubated at 4 °C overnight. Samples were then washed twice with sterile PBS and incubated in a respective second conjugated antibody solution, comprising anti-mouse Alexa Fluor 594 A-11005 (1:500) and anti-rabbit Alexa Fluor 488 A-11008 (1:500) dissolved in buffer (PBS 1X, 3% BSA, 0.3 Triton X-100), for 1 h at RT in the dark. Finally, specimens were washed thrice with sterile PBS and mounted in Fluoromount-G^®^ with DAPI (Thermo Fischer Scientific) for 24 h at RT in the dark. The visualization of NET structures based on co-localized extracellular DNA staining and histone- and NE-derived signals, was achieved by using a confocal inverted microscope known as Stellaris 5^®^ (Leica, Wetzlar, Germany).

### 2.5. Extracellular DNA-Based Quantification of Cell-Free and Anchored NETs Phenotypes Induced by FhAg Under Hypoxia

For the determination of cell-free and anchored NETs, the protocol of [42] was followed. It indicates that extruded NETs can be divided into two distinct forms: (i) cell-free NETs, which are NETs released away from and without contact with ruptured PMNs, and (ii) anchored NETs, which are NETs that are released from but still anchored to ruptured PMNs. Briefly, sheep PMNs (*n* = 4; 5× 10^5^, in duplicates) were resuspended in sterile serum-free RPMI 1640 media without phenol red (Sigma-Aldrich) and co-cultured for 180 min with soluble *Fh*Ag (100 μg/mL) in hypoxic conditions (5% O_2_, 94% N_2_, 5% CO_2_) at 37 °C using a hypoxia incubator chamber (Stemcell Technologies^®^) in 96-well plastic flat-bottom plates (Greiner, Kremsmünster, Austria). After incubation, 100 µL of culture supernatant was collected from each well and transferred to another well for the quantification of cell-free NETs by PicoGreen^®^-based fluorometric measurements (Thermo Fisher Scientific, Waltham, MA, USA). Then, fresh medium was added to each well after the removal of the supernatant and PicoGreen^®^-based fluorometric measurements were likewise used to detect anchored NETs attached to the bottom of culture wells. For both sampling methods, a 1:200 dilution of PicoGreen^®^ (Invitrogen, Waltham, MA, USA) in 10 mM Tris base buffered with 1 mM EDTA was added to each well (50 μL), and then extracellular DNA was detected and quantified using PicoGreen^®^-derived fluorescence intensities via spectrofluorometric analysis. This involved using an automated multiplate monochrome reader (Varioskan Flash^®^; Thermo Scientific) at 484 nm excitation/520 nm emission. As controls, we used PMNs alone, *Fh*Ag alone (100 μg/mL), and PMNs treated with Triton X (0.1%; Sigma-Aldrich, St. Louis, MO, USA) for the assessment of total DNA and we used PMNs exposed to calcium ionophore A23187 (5 µM; Sigma-Aldrich) as the positive control. For comparison purposes, the same experimental settings were used under hyperoxic conditions (21% O_2_, i.e., the most commonly used lab oxygen condition worldwide).

### 2.6. Determination of NADPH Oxidase (NOX) Inhibition on NET Formation

In addition, given the fact that ROS production has been observed to increase upon the exposure of ovine PMNs to *Fh*Ag [38], here, we also determined the blocking of NOX complex formation using the NOX-specific inhibitor DPI. In brief, ovine and bovine PMNs, DPI (10 μM, Sigma-Aldrich), and *Fh*Ag (100 μg/mL) were incubated for 180 min at 37 °C under previously indicated hypoxic (5% O_2_) and hyperoxic conditions (21% O_2_).

### 2.7. Flow Cytometric Analysis of FhAg-Induced CD11b Surface Expression on Ovine PMNs

Ovine PMNs were incubated with 100 µg/mL of *Fh*Ag for 15 and 30 min at 37 °C in HBSS containing 0.9 mM CaCl_2_. For the positive controls, PMNs were treated with platelet-activating factor (PAF 100 nM, 15 min, 37 °C; Calbiochem, Darmstadt, Germany). For negative controls, non-treated PMNs were used. After incubation, cells were centrifuged (300× *g*, 20 °C, 6 min); resuspended in 200 μL HBSS; incubated with anti-CD11b antibodies, coupled with the FITC clone M1/70, for 20 min in the dark (BD Pharmingen); and washed at 300× *g* and 20 °C for 6 min. Thereafter, cells were analyzed using a BD FACSCanto II^®^ cytometer (BD Biosciences, Franklin Lakes, NJ, USA). Data were displayed as plots of forward versus side light scatter. The mean fluorescence of FITC was determined by using BD FacsDiva v 6.1.3 (BD Biosciences).

### 2.8. Isolation of FhAg- and A23187-Induced NETs

The isolation of NETs was performed as previously described by [43] with some modifications. Briefly, 1.5 × 10^6^ bovine PMNs/well (*n* = 4) were seeded in 12-well culture plates and stimulated with either 100 μg/mL *Fh*Ag or using the calcium ionophore and PMN activator A23187, a potent inductor of NETs, according to [44]. This was the positive control for NET formation and was performed in a concentration of 5 µM (Sigma-Aldrich) for 3 h (37 °C, 5% CO_2_). After incubation, the medium was carefully aspirated, and wells were washed twice with 1 mL of PBS. Then, 400 μL of AluI (4 U/mL, New England Biolabs, Ipswich, MA, USA) was added and plates were incubated for 20 min at 37 °C and 5% CO_2_. Thereafter, samples were recovered and centrifuged for 5 min at 300× *g* to remove cells and debris. NET preparations were immediately stored at −80 °C until further quantification and use. The DNA content of NET preparations was estimated by Quant-iT PicoGreen^®^ (Thermo Fisher Scientific). Briefly, 2 μL of each NET sample was mixed using 98 μL of TE buffer (1 M Tris pH 7.4; 0.5 M EDTA pH = 8.0) and incubated for 5 min at RT, protected from light. Afterwards, the DNA content was quantified in a Varioskan Flash^®^ fluorescence automated multiplate reader (Thermo Scientific, USA), applying exposition/emission wavelengths of 480/520 nm, respectively. All DNA measurements were performed in duplicate. A standard λ-DNA curve was used to interpolate the DNA concentration of the samples. The preparation of pure NETs resulted in concentrations of 94.5 ng DNA/mL for *Fh*Ag-NETs and 113.5 ng/mL for A23187-NETs. Overall, an average of 21.4 ng DNA per 10^6^ PMNs was obtained.

### 2.9. Estimation of FhAg-Induced NETs Cytotoxicity in Exposed Hepatic Cells In Vitro

For the estimation of cytotoxic effect of *Fh*Ag-induced NETs, we used a concentration of 3.3 ng DNA/mL, working according to [43]. Murine hepatocytes of the liver cell line MSCS10 (CD-1; Gibco Waltham, MA, USA) were seeded on 96-well black plates (SPL Life Science, Pocheon, Republic of Korea) in a concentration of 80,000 hepatocytes per well in triplicate. Then, hepatocytes were treated with NETs isolates obtained from PMNs exposed to 100 μg/mL *Fh*Ag and 5 µM A23187 for 1.5 h at 37 °C in a humidified incubator with a 5% CO_2_ atmosphere. Since core histones are the most abundant proteins of extruded NETs (i.e. 70% of all NET-associated proteins) and as H2A represents the 26.9% of the total NET protein content [32], we also determined whether a major single NET component, such as H2A, can also induce cytotoxicity and damage in the hepatocyte culture in concentrations of 200 μg/mL (Sigma-Aldrich), working according to [43]. As controls, DNA from non-treated PMNs (control DNA) was used, as were unexposed hepatic cells marked with Sytox Orange (cells + SO). After exposure, the medium was removed and cells were analyzed to assess cytotoxic effects via a dead cell stain (5 μM Sytox Orange^®^; Thermo Fisher Scientific) diluted in modified ECGM medium (10 min, RT, in the dark). Fluorescence intensity was estimated at 574/570 nm excitation and emission wavelengths were expressed as relative fluorescence units (RFUs) using a multiplate monochrome reader (Varioskan Flash^®^; Thermo Scientific).

#### Statistical Analyses

Statistical analyses were performed using GraphPad Prism^®^ version 9.5.1, obtained from GraphPad^®^ Software, San Diego, CA, USA. For the comparison of two or more groups, we applied one-way ANOVA with Tukey post hoc test multiple comparisons. Statistical significance was defined at *p* < 0.05.

## 3. Results

### 3.1. Immunofluorescence Analysis Confirmed Extrusion FhAg-Induced NETs Under Hypoxic Conditions In Vitro

**Figure 1:**
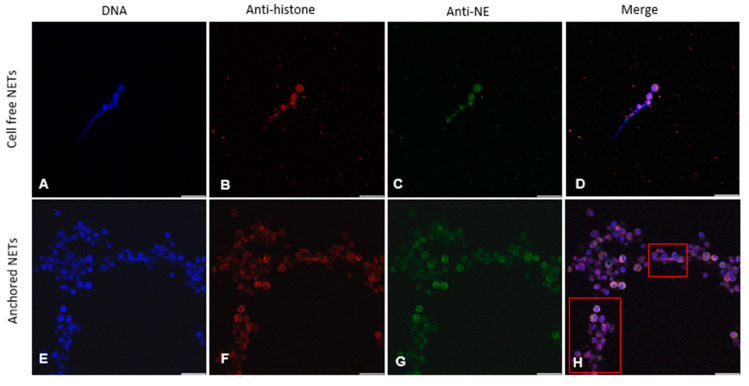

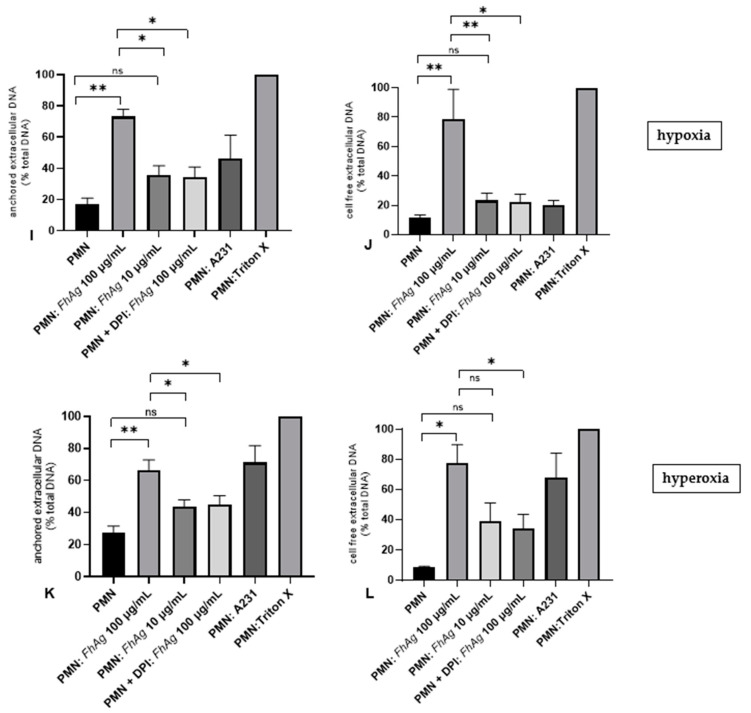
***Fasciola hepatica* antigen (*Fh*Ag)-induced neutrophil extracellular trap (NET) formation under hypoxia (5% O_2_) and hyperoxia (21% O_2_), analyzed via fluorescence microscopy, and PicoGreen^®^-derived fluorescence intensities.** Fluorescence microscopy analysis demonstrates the co-localization of DNA (**A**,**E**; blue), pan-histones (H1, H2A/H2B, H3, H4) (**B**,**F**; red), and the neutrophil elastase (NE) (**C**,**G**; green) of ovine PMNs subjected to 100 μg/mL *Fh*Ag, indicating a spread NET (*spr*NETs) phenotype in both cell-free and anchored NETs. The depiction of the overlay of the three channels (**D**,**H**). PicoGreen^®^-derived fluorescence intensity analysis shows the quantification of anchored NETs under hypoxia (**I**,**J**), demonstrating a significant increase compared with control PMNs at 180 min of incubation (*p* < 0.01; **I**) and an increase in the release of cell-free NETs, induced by 100 μg/mL *Fh*Ag at 180 min incubation, when compared to control PMNs (*p* < 0.01; **J**). The inhibition of the NADPH oxidase (NOX) complex with the NOX inhibitor diphenylene iodonium (DPI) resulted in a significant reduction in anchored NETs (*p* < 0.05; (**I**)) and cell-free NETs (*p* < 0.05 (**J**)) under hypoxic conditions. For comparison purposes, the same settings were used under hyperoxia (21% O_2_ (**K**,**L**)), indicating the extrusion of anchored NETs (*p* < 0.01; (**K**)) and cell-free NETs (*p* < 0.05 (**L**)). As controls, we used PMNs alone, PMNs treated with Triton X (0.1%) for the assessment of total DNA, and PMNs exposed to A231 (5 µM) as a positive control. For statistical analyses, ANOVA with multiple comparisons was applied. Differences were regarded as significant at a level of *p* ≤ 0.05 (*); *p* ≤ 0.01 (**); ns: not significant.

Classical components of NETs extruded under hypoxic environmental conditions were investigated via immunofluorescence microscopy analyses. Ovine PMNs co-cultured with soluble *Fh*Ag were used to validate the traditional hallmarks of mammalian-derived NETs, proving the presence of extracellular chromatin (Figure 1A,E, blue) and pan-histones [H1, H2A/H2B, H3, H4] (Figure 1B,F, red) with NE (Figure 1C,G, green). Thus, the co-localization of extracellular DNA with histones and NE confirmed the presence of typical NET proteins associated with the chromatin released from ruptured PMNs (Figure 1D,H, merge), thereby confirming the formation of NETs under hypoxic conditions.

### 3.2. Hypoxic Conditions Enhance the Formation of Anchored and Cell-Free NET Phenotypes Induced by FhAg

In addition, during fasciolosis, the flukes must confront low oxygen levels in the liver and the bile [20]. Therefore, in order to be closer to the in vivo physiological environment, the NETosis quantification of anchored and cell-free NETs induced by *Fh*Ag was performed in hypoxia. We observed that PMNs underwent NETosis, leading to a significant increase in anchored NETs (*p* < 0.01, Figure 1I), whose formation was induced by 100 μg/mL *Fh*Ag, after 3 h of incubation. Meanwhile, cell-free NETs were likewise released upon exposure to 100 μg/mL *Fh*Ag, and their increase was also significant (*p* < 0.01, Figure 1J). In addition, both the presence of anchored and cell-free NETs increased in a dose-dependent manner (Figure 1I,J). However, low doses of *Fh*Ag (10 μg/mL) did not trigger a significant increase when compared to PMNs in control media (Figure 1I,J). As a control, in parallel settings, the released of anchored and cell-free NETs in hyperoxia (21% O_2_) was analyzed. We observed significant increase in both cell-free and anchored NETs upon exposure of PMNs to 100 μg/mL *Fh*Ag (*p* < 0.01 anchored, Figure 1K; *p* < 0.05 cell-free, Figure 1L). Interestingly, under these experimental settings, calcium ionophore A231 proved to be a suitable positive control for NET induction in ovine PMNs. Immunofluorescence images of anchored NETs are also displayed in Figure 1D,H.

### 3.3. NADPH Oxidase (NOX) Inhibition Significantly Reduces Extrusion of Anchored and Cell-Free NETs Under Hypoxia

Meanwhile, the inhibition of the NOX complex with the NOX-specific inhibitor DPI resulted in a significant reduction in both phenotypes, i.e., anchored NETs (Figure 1I, *p* < 0.05) and cell-free NETs (Figure 1J, *p* < 0.05), after 180 min incubation with *Fh*Ag (100 μg/mL) under hypoxic conditions (5% O_2_). Likewise, treatment with DPI under hyperoxic conditions (21% O_2_) also resulted in a significant reduction in anchored and cell-free NETs (Figure 1K,L; *p* < 0.05). Data are displayed as data normalized to the total DNA released from cells (Triton X control) in each case.

### 3.4. FhAg-Triggered NETs (FhAg-NETs) Are Cytotoxic for Hepatic Cells

**Figure 2:**
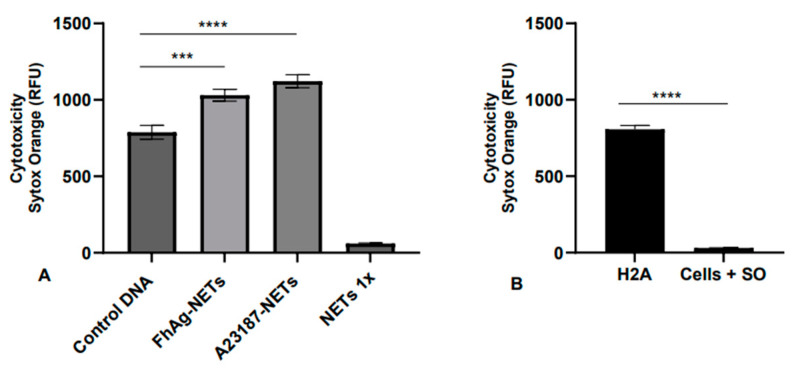
***Fh*Ag-derived NETs (*Fh*Ag-NETs) and A23187-derived NETs (A23187-NETs) are cytotoxic for hepatic cells.** Hepatic cells (MSCS10) were treated with NET isolates obtained from PMNs exposed to 100 μg/mL *Fh*Ag and 5 µM A23187 for 1.5 h at 37 °C in a humidified incubator with 5% CO_2,_ resulting in significantly enhanced cell death compared to the DNA control (*Fh*Ag-NETs *p* = 0.001; A23187-NETs *p* < 0.0001; (**A**)). As a control for cell death, H2A was used in concentrations of 200 μg/mL, which resulted in significantly enhanced cell death (*p* < 0.0001; (**B**)). As a control, DNA from non-treated PMNs (control DNA) was used, as were unexposed hepatic cells marked with Sytox Orange (cells + SO). After exposure time, the medium was removed, and cells were analyzed for cytotoxic effects via dead cell staining (5 μM Sytox Orange^®^), with results expressed as relative fluorescence units (RFUs). Differences were regarded as significant at a level of *p* ≤ 0.001 (***); *p* ≤ 0.0001 (****).

For the estimation of cytotoxic effect of *Fh*Ag-NETs on hepatic cells, we determined cell death on hepatocytes by using a live/dead staining with Sytox Orange^®^ (Thermo Fisher Scientific, Waltham, MA, USA), which is widely used as a cell death marker. Overall, treatments of hepatic cells (MSCS10) with NET preparations obtained from ovine PMNs, stimulated with either parasite antigens (*Fh*Ag-NETs) or the calcium ionophore A23187 (A23187-NETs) used as the positive control, resulted in significantly enhanced cell death compared to the DNA control (*Fh*Ag-NETs *p* = 0.001; A23187-NETs *p* < 0.0001; Figure 2A), with a stronger reaction showed by A23187-NETs with respect to *Fh*Ag-NETs. As a positive control for cell death, we used a major single NET component H2A at 200 g/mL, which resulted in significantly enhanced cell death (*p* < 0.0001; Figure 2B) when compared to the control. Overall, here, we demonstrate that *Fh*Ag-NETs are cytotoxic for hepatocytes.

### 3.5. FhAg Exposure Enhances Matrix Metalloprotease 9 (MMP-9) Release in Ovine- but Not in Bovine PMNs

**Figure 3:**
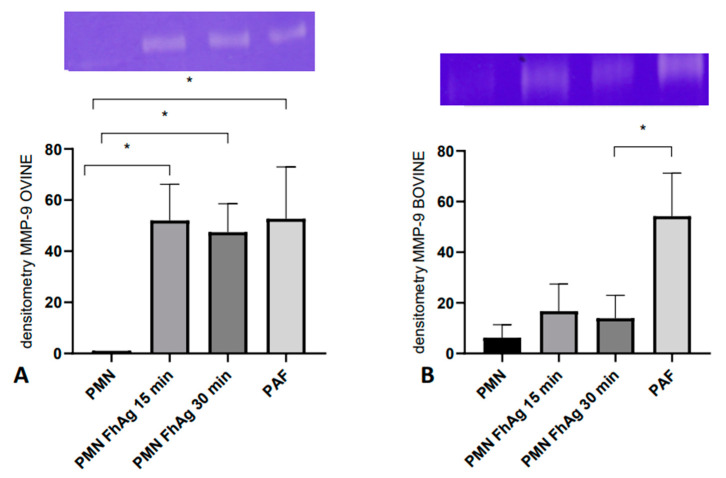
**MMP-9 release of ruminant-derived PMN granule contents after *Fasciola hepatica* antigen (*Fh*Ag) exposure.** Ovine PMNs (**A**) and bovine PMNs (**B**) were exposed to 100 μg/mL *Fh*Ag for 15 and 30 min of incubation at 37 °C and the supernatants were analyzed for MMP-9 activities via zymography in gelatin-containing polyacrylamide gels and consecutive densitometric estimations. Co-culturing ovine PMNs with *Fh*Ag led to a significant increase in MMP-9 release (*p*  < 0.05; (**A**)) at both time points tested. In contrast, bovine PMNs did not significantly increase the MMP-9 release (**B**). Recombinant MMP-9 was used as an internal standard. The stimulation of PMNs with PAF (100 nM) served as the positive control. For statistical analyses, ANOVA with multiple comparisons was applied. Differences were regarded as significant at a level of *p* ≤ 0.05 (*).

The enzymatic activity of MMP-9 was analyzed on PMN supernatants, where the co-culturing of ovine PMNs with *Fh*Ag led to a significant increase in MMP-9 release (*p*  <  0.05; Figure 3A) at both time points tested (15 and 30 min), indicating that tertiary granules or MMP-9 may also be involved in *Fh*Ag-triggered NETosis and tissue damage in vivo. On the contrary, for bovine PMNs, no significant increase in MMP-9 release was observed upon exposure to *Fh*Ag (Figure 3B). As expected, stimulation with PAF revealed to be a reliable positive control for MMP-9 release in both the ovine and bovine systems (*p*  < 0.05).

### 3.6. Exposure of Ovine PMNs to FhAg Did Not Increase Surface Expression of CD11b

**Figure 4:**
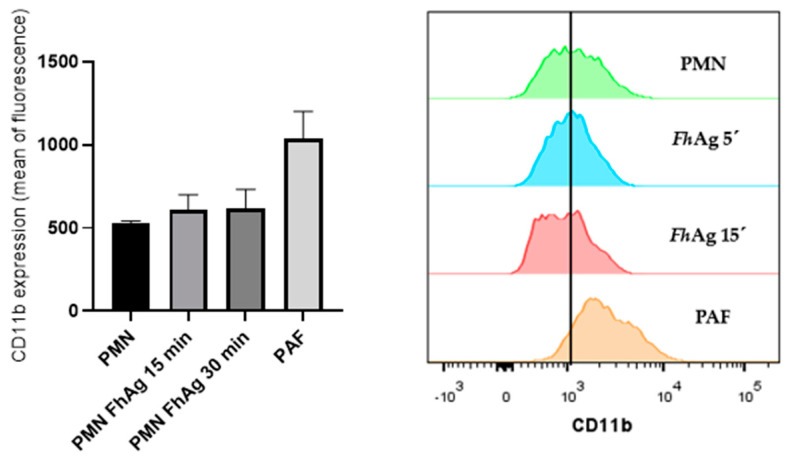
**The *Fasciola hepatica* antigen (*Fh*Ag) does not enhance CD11b surface expression on ovine PMNs.** Ovine PMNs were incubated with 100 µg/mL of *Fh*Ag for 15 and 30 min at 37 °C and subjected to flow cytometric analysis by using antibodies directed against CD11b. The histograms represent the shift in the mean fluorescence of the PMN population analyzed and are representative of the experiments performed. Non-exposed PMNs in the plain medium served as negative controls. Stimulation with PAF (100 nM) served as a positive control. Each bar represents the arithmetic means ± SEM of three PMN donors (*n* = 3).

Since no data are available on PMN-derived receptors being involved in *F. hepatica*-mediated NETosis, here, we analyzed whether *Fh*Ag exposure would affect CD11b surface expression on ovine PMNs. Applying flow cytometric analysis methodology, no difference was detected in terms of CD11b surface expression compared to the PMN control (Figure 4A,B), suggesting other receptors were activated during *Fh*Ag-mediated NETosis.

## 4. Discussion

In the pathogenesis of fasciolosis, newly excysted juvenile flukes must traverse the duodenal wall to enter the peritoneal cavity, where they migrate through and finally penetrate the liver Glisson capsule in order to invade the hepatic parenchyma [9], moving to the bile ducts where they mature into adults [12]. Throughout its liver migration, *F. hepatica* is continually exposed to hostile conditions within the host, including cellular barriers, innate and adaptive immune cells, complement proteins, antibodies, antimicrobial peptides, pro-inflammatory cytokines, chemokines, and various other soluble factors [3,37]. We have previously demonstrated the ability of *Fh*Ag to trigger ovine NETosis, intracellular ROS production, chemotaxis in vitro, and leukocyte infiltration with the concomitant formation of NETs in vivo [38]. These findings provide new insights on the pathogenesis of liver fluke disease in the ovine system. Along these lines, studies on trematode *Opisthorchis viverrini* crude antigen-induced NET formation showed that the excessive production of pro-inflammatory mediators from NETs may lead to the excessive injury of surrounding tissues, resulting in hepatobiliary abnormalities, and may eventually lead to very severe complications such as cholangiocarcinoma [35]. In addition, PMNs operate in infected and inflamed tissues, which can be profoundly hypoxic (1–5% O_2_). Accordingly, the neutrophilic infiltration of hypoxic tissues characterizes a myriad of acute and chronic infections as well as pro-inflammatory diseases. PMN granule products present in NETs are implicated in causing collateral tissue damage in these scenarios [45,46,47]. For instance, in hepatic cancer, intratumoral hypoxia accentuated NET formation in vivo [48]. Here, we demonstrate for the first time that NETosis in response to *Fh*Ag is also triggered under hypoxia in vitro, displaying typical NET-associated characteristics. This was confirmed by immunofluorescence analyses via co-localization experiments, observing the extrusion of extracellular DNA from ovine PMNs coated with H1, H2A/H2B, H3, H4, and NE. These data corroborate previous data from the same analyses performed in hyperoxia (i.e., 21% O_2_), indicating that PMNs incubated with adult *Fh*Ag receive sufficient stimuli to generate classical NETs [38]. These findings are consistent with those of similar in vitro studies that used soluble parasite antigens to assess PMN-mediated immune responses, suggesting that parasite-specific antigens may enhance uptake or improve the availability of potential pathogen-associated molecular patterns (PAMPs) to PMNs [49]. Interestingly, here, we used total soluble antigens that may differ from the excreted/secreted (E/S) antigens that should be available in the infected organs in vivo. Furthermore, the quantification of anchored and cell-free NETs under hypoxia was performed by PicoGreen^®^-derived fluorescence analysis, observing the release of both cell-free and anchored NETs in ovine PMNs exposed to *Fh*Ag. Interestingly, this process proved to be ROS-dependent since a significant reduction in NET formation was observed following treatments of PMNs with NOX-inhibitor DPI, confirming the key role of these enzymes in *F. hepatica*-induced ovine NETs. Additionally, it has been demonstrated that ROS production is upregulated in a CD11b (CR3)-dependent manner in bovine PMNs [50], and that this integrin receptor has been involved in apicomplexan parasite-mediated NETosis [27] and in fungi- and bacteria-induced NETosis, including *Candida albicans* [51] and *Mannheimia haemolytica* [52]. However, the exposure of ovine PMNs to *Fh*Ag did not result in a significant upregulation of this integrin receptor. This could be an effect of the activation of other surface receptors or transcription factors involved in *F. hepatica*-induced NETosis in the ovine system. Since PMN degranulation represents a significant effector mechanism against *F. hepatica* stages, both in the ovine and bovine system, and specifically in metalloproteinase (MMP) release from tertiary (gelatinase) granules, we tested whether MMP-9 is released in response to *Fh*Ag exposure in ovine and bovine PMNs. Thus, in this study, we performed zymographic analyses of PMN supernatants exposed to *Fh*Ag and demonstrated that ovine PMN stimulation with *Fh*Ag led to a significant increase in MMP-9 release. This finding was in contrast to bovine PMNs, where no significant increase in MMP-9 release was observed under same experimental settings. Other investigations demonstrated that the apicomplexan parasite *Eimeria bovis* significantly increased MMP-9 release into bovine PMN supernatants [27]. Overall, here, we confirm differences in the degranulation contents of two highly productive ruminant species frequently exposed to *F. hepatica* infections, demonstrating the complexity of species-specific parasite interactions, expressed as different degrees of susceptibility or species resistance upon encountering the same endogenous parasite.

Corresponding to our in vitro data, the evidence of NETs being released in the hepatic parenchyma of sheep with fasciolosis confirmed the relevance of this as an early host innate immune reaction in vivo. Additionally, different phenotypes of *F. hepatica*-triggered spread NETs (*spr*NETs) and diffuse NETs (*diff*NETs) were demonstrated in vitro and in vivo [38], suggesting that NET formation in hepatic parenchyma and/or bile ducts might play a pivotal role in the pathogenesis of either acute or chronic fasciolosis, particularly in hepatic parenchyma areas with major leukocyte infiltration. NET release induced by the apicomplexan *Besnoitia besnoiti* has been proved to induce endothelial dysfunction, cell death, and endothelium damage in vitro [43], thereby also proving the adverse NETosis-derived effects on blood vessels. Here, we estimated the cytotoxic effects of extruded NETs on hepatic cells by using a fluorescent cell death marker, observing that the treatment of hepatic cells with ovine *Fh*Ag-NETs significantly enhanced hepatocyte death compared to the control. Consequently, we hypothesize that NET formation in response to *F. hepatica* may contribute to exacerbated hepatic damage, associated with the migration of juvenile and adult flukes into the bile ducts. Accordingly, our results strongly suggest that tertiary granules or MMP-9 alone may also be involved in *Fh*Ag-triggered NETosis and tissue damage in vivo. The functional role of MMP-9 and other molecules/enzymes in ruminant NETosis will be investigated further. The same holds true for investigations on the transient receptor potential vanilloid-type 4 (TRPV4) cation channel, widely expressed in all tissues and PMNs, and its function as mechanosensitive Ca^2+^ channel against this zoonotic-relevant trematode.

## 5. Conclusions

During fasciolosis, the parasitic zoonotic trematode *F. hepatica* must tolerate low oxygen levels in the liver parenchyma and/or bile ducts. The same holds true for mammalian PMNs which can also operate in infected and inflamed tissues with profound hypoxic conditions between 1 and 5% O_2_. Here, we demonstrate that NET formation in response to *Fh*Ag occur under these adverse oxygen conditions, which are very close to the in vivo situation. In these adverse environments, the NETs released also play a role in contributing to tissue damage in infection sites, as previously reported. In the present study, we demonstrated that extruded *Fh*Ag-NETs are cytotoxic for hepatic cells in vitro, thereby supporting our hypothesis regarding the possible contribution of NETs to the pathogenesis of fasciolosis in vivo. In addition, PMN-derived tertiary granule contents such as MMP-9 are also released in response to this parasite, and its role in tissue damage will be further investigated, as will other ruminant receptors activated by *F. hepatica* in ovine and bovine PMNs.

## Data Availability

The data that support the findings of this study are available from the corresponding author upon reasonable request.
